# Darkness increases the population growth rate of the poultry red mite *Dermanyssus gallinae*

**DOI:** 10.1186/s13071-019-3456-1

**Published:** 2019-05-07

**Authors:** Chuanwen Wang, Yuyun Ma, Yu Huang, Shanchun Su, Lianyu Wang, Yanyan Sun, Qiang Wan, Hao Li, Shudong Zhang, Øivind Øines, Baoliang Pan

**Affiliations:** 1 College of Veterinary Medicine, China Agricultural University, Beijing, 100193 China; 2 Animal Disease Prevention and Control Centre of Pinggu District, Beijing, 101200 China; 3 Norwegian Veterinary Institute, PO Box 750, Oslo, Norway

**Keywords:** *Dermanyssus gallinae*, Poultry red mite, Darkness, Reproduction

## Abstract

**Background:**

The poultry red mite (PRM), *Dermanyssus gallinae*, is one of the most economically deleterious ectoparasites affecting egg-laying hens worldwide. It may be possible to control *D. gallinae* populations by manipulating lighting regimes within poultry units. However, no studies have clearly shown the effects of darkness on the population growth rate of *D. gallinae*.

**Methods:**

The effect of darkness on the population growth rate of *D. gallinae* was investigated, together with the first description of the molecular identity of the mite from China. Mite variables under two lighting regimens (1:23 h L:D and 12:12 h L:D) were compared, including number of mites and eggs, survival and feeding rates, engorgement, oviposition, hatchability and the life-cycle of *D. gallinae.*

**Results:**

The results showed that the number of mites (13,763 ± 956) and eggs (5424 ± 317) in the rearing system with prolonged darkness of 1:23 h L:D at 4th week were 2.4- and 3.6-fold higher than those under a conventional lighting regimen of 12:12 h L:D, respectively. The feeding rates of mites under prolonged darkness ranged from 36.7 ± 1.1% to 52.0 ± 7.0%, which were significantly higher than those under conventional lighting regimen (ranging from 22.6 ± 1.9% to 37.3 ± 1.6%). The mean weight of engorged females (0.26 ± 0.01 mg) and the mean number of eggs per female (on average 5.87 ± 0.36) under prolonged darkness were significantly higher than those under conventional lighting regimen (0.22 ± 0.01 mg and 3.62 ± 0.31, respectively). However, the survival rate ranging from 98.07 ± 0.10% to 98.93 ± 0.19%, hatchability of 97.93 ± 0.01% and the life-cycle of *D. gallinae* (9 days) was not affected by the lighting period.

**Conclusions:**

Our findings demonstrated that prolonged darkness significantly promoted the proliferation levels of *D. gallinae*, resulting in increased number of mites and eggs in the rearing system. The promoted population growth of *D. gallinae* was found to be related to the increased feeding rate, engorgement level and oviposition level of mites under prolonged darkness. The egg hatchability, the survival rates and the duration of life-cycle of *D. gallinae* were not affected by the light regimes.

## Background

The poultry red mite (PRM), *Dermanyssus gallinae*, is the most significant parasitic threat to egg-laying hens, worldwide [1]. This species is reported from layers in many parts of the world, including the USA, Europe, Japan and China [2–4]. This blood-sucking ectoparasite causes widespread problems in egg production and challenges animal health, the animal welfare and raises an increased public health concern due to its capacity of being a vector of various animal and zoonotic pathogens [1]. During the night, *D. gallinae* feeds on hens by sucking blood, causing restlessness and stress, fatigue, reduced egg quality and egg production, increased anemia and in severe cases, mortality [5]. It has been reported that *D. gallinae* serves as a vector for a range of different poultry pathogens and zoonoses such as *Salmonella* spp., suggesting a potential risk to human health [6]. In Europe, it has been estimated that this parasite causes about €231 million in annual losses to European egg producers [7]. Furthermore, it has been acknowledged that *D. gallinae* can affect the health and wellbeing of persons working with poultry, reportedly causing avian mite dermatitis/gamasoidosis; manifested as itching, allergic reactions, and by inflammation of the skin [8].

*Dermanyssus gallinae* has a high reproductive capacity and rapid growth, e.g. the life-cycle can be completed in just over a week [9], and so large populations of red mites can quickly build up in the poultry houses. *Dermanyssus gallinae* are highly resistant to starvation and can survive for up to eight months under natural conditions without a blood meal available [2]. Under conventional photoperiod conditions, the mites spend most of their time off a host, living in cracks and crevices within the poultry house and equipment, leaving their hiding place only to attack hens and briefly suck blood [2]. Their high reproductive capacity, location off the host often hiding in cracks and crevasses, with their migratory behavior, lead to difficulties in the control of *D. gallinae* using traditional approaches such as sanitary intervention and chemical distribution. Control measures have concentrated on treating the premises rather than the individual birds, and have primarily relied on the use of insecticides. Recently, fluralaner solution distributed through the hens drinking-water, showed a promise for the use of chemicals to lower infestation levels in flocks [10]. However, with increased regulation associated with the use of chemicals for food production, and chemical resistance towards various chemicals becoming common, alternative methods of control are needed.

It is generally believed that *D. gallinae* attacks hens at night and remains hidden during the day [5]. A better understanding of the effects of light/darkness on the mites’ behavior could provide an insight into the biological characteristics of *D. gallinae*, a step towards the development of control strategies and a method for optimizing conditions for rapid rearing *D. gallinae* in laboratory.

A number of previous studies conducted in laboratory conditions or on layer farms have attempted to do this with varying degrees of success [11–16]. Initially, Harrison [15] suggested that light was one of the key factors influencing activity of *D. gallinae*. Kirkwood [13] further reported that more mites fed in darkness than in light. Under laboratory conditions, Zoons [11] and Stafford [12] observed that short-cycle intermittent light/dark periods could markedly reduce *D. gallinae* numbers compared with more standard lighting regimens, probably by disrupting the mites’ normal nocturnal feeding cycle, although the exact reasons remain unclear [11, 12]. Kilpinen [14] observed that when subjected to light in the presence of CO_2_, mite would freeze, possibly as a behavioral response to avoid predation. Sokół et al. [16] conducted a study under field conditions to evaluate the effects of two photoperiods on the behavior of mites on layer farms: one light program with an 8-hour night phase and a 16-hour day phase, the other comprising 2 hours (h) of darkness and 4 h of light applied alternately during the day. They found that the alternating light and dark cycle led to few trapped mites. The speculated reason was that this lighting program motivated the mites to move constantly and the stress caused by these frequent light changes disrupted reproduction while the darkness in the layer house seemed more likely to stimulate *D. gallinae* reproduction, than light. Therefore, the potential for poultry red mite infestations control by intermittent lighting, may contribute to reduce this problem in egg-laying hens. It has been found that intermittent lighting also provided other welfare benefits, including a reduction in hen mortality, the alleviation of heat stress, an increase in docility and a reduction in cannibalistic behavior [17]. However, the current regulations should be seriously considered. For example, the current EU legislation requires a statutory 8-h dark period to ensure that the hens may rest and to avoid problems such as immunodepression and ocular anomalies [5], which makes it difficult to envisage how intermittent lighting regimens could be employed in practice. One possible solution was proposed by Sparagano et al. [5] that intermittent lighting could perhaps be used during normal light periods with (presumably) fewer welfare implications.

Although it is demonstrated that darkness was crucial to the reproduction and survival of *D. gallinae*, and there is potential to control this parasite *via* lighting rhythm. However, no studies have investigated the detailed effect of darkness on mite reproduction and the population dynamics of *D. gallinae* under prolonged dark conditions. Such studies can give basic knowledge of the effect of light/darkness on the proliferation levels of *D. gallinae*, providing an insight into the biological characteristics of this species, a step towards the development of control strategies *via* changing the lighting regimes and a method for optimizing conditions for rearing *D. gallinae* in laboratory.

An *in vivo* rearing system for *D. gallinae* under laboratory conditions has been developed previously by our group [18]. In this system, experimental conditions, e.g. light intensity and time can be well controlled, and various mite variables, e.g. mite stage numbers and egg production can be recorded accurately. The work described here focused on fundamental questions of the effect of darkness on the population growth rate of *D. gallinae*. We evaluated the effect of two photoperiods: conventional light regime of 12 h light followed by 12 h darkness (12:12 h L:D) and a prolonged darkness photoperiod receiving only 1 h light followed by 23 h darkness (1:23 h L:D) on the population growth rate of *D. gallinae*. We also evaluated the effect of darkness on the mite’s survival and feeding rates, oviposition and the egg hatchability as well as the duration of life-cycle of *D. gallinae* under the two photoperiods. The mechanism of darkness increasing the reproduction rate of *D. gallinae* was discussed.

## Methods

### *Dermanyssus gallinae* used in the experiments

*Dermanyssus gallinae* were taken from a laboratory culture, which was originally collected from a commercial poultry farm in Pinggu District of Beijing in China, and since then was kept in the laboratory by feeding on chicks [18], named as CBP-1. To confirm the identity of the isolated strain of mite (CBP-1), molecular identification was carried out following previously described PCR-method [19]. Briefly, three individual adult mites were crushed, and DNA was extracted following the tissue protocol for the TIANamp Genomic DNA Kit (Tiangen Biotech Co., Ltd, Beijing, China). Two regions of DNA, parts of the mitochondrial cytochrome *c* oxidase subunit 1 (*cox*1) gene and parts of the internal transcribed spacer (ITS) were amplified by PCR, and sequenced. The primers used were those described by Øines & Brännström [19]. For *cox*1 amplification, forward primer (FCOIDG) and reverse primer (RCOIDG) amplified a 737-bp fragment of the 5′ region of the cDNA predicted to be *cox*1. For amplification of the ITS fragment, forward primer (ITS-1) and reverse primer (ITS-RDG) amplified a region between ITS1 and ITS2 containing the 5.8S ribosomal subunit gene. PCR cycling conditions were as follows, initial denaturing at 95 °C was performed for 2 min, followed by 35 cycles of denaturing at 95 °C for 30 s, annealing at either 46 °C for 30 s or 47 °C for 2 min, and elongation at 72 °C for 1 min, and final elongation at 72 °C for 10 min. The PCR-products were examined on 1.5% agarose gels, with a DNA ladder (Tiangen Biotech Co., Ltd) included. The amplified PCR products were sequenced by a commercial company (Sangon Biotech Co., Ltd, Shanghai) using ABI 3730XL DNA Analyzer. ITS and *cox*1 sequences from CBP-1 individuals were edited using ChromasPro/ or Contig Express (Vector Nti, Thermo Fisher Scientific, Waltham, USA). For *cox*1 sequences, primer sites were manually removed and ORF checked on the 672 bp sequence to eliminate sequence error, and to verify the reading frame of the resultant sequences (+1).

### An *in vivo* feeding system of *D. gallinae*

The *in vivo* feeding method was based on a protocol described previously by our group [18] and is briefly described herein: three 5-day-old chicks were kept in the cage as host animals of *D. gallinae*, which were provided with water and feed *ad libitum*. The animals were kept in a metal cage, which was placed inside a plastic storage box with petroleum jelly applied as continuous lines across the top four edges to function as a barrier, preventing the escape of *D. gallinae* from the storage box. The storage box was placed over a tray filled with water to prevent *D. gallinae* migrating out of the feeding system. Traps consisted of a plastic centrifuge tube (10 ml) and a 6 × 6 cm piece of a disposable mask which placed inside the tube. Traps (*n* = 12 per cage) were fixed at the aggregation site of mites around the bottom of the cage. The total feeding devices were placed in the artificial climate incubator (RXZ-500B-LED, Ningbo Jiangnan Instrument Factory, China) at 30 °C, 75% relative humidity (RH), and with a light intensity of approximately 3600 lux. And the light regimen was 12:12 h L:D. The chicks were fed with a commercial poultry feed (Feed Industry Centre, Ministry of Agriculture), and were housed under a protocol approved by Laboratory Animal Institute of China Agricultural University within Beijing Animal Use and Care Association (Approval No.: CAU20180408-2).

### The population dynamics of *D. gallinae* under prolonged darkness

In this trial, the chicks and *D. gallinae* were kept on prolonged darkness photoperiod of 1:23 h L:D. The time of 1 h light was included to ensure that the mites returned to the monitoring traps at each observation, enabling mite counting. The cages and the chicks were confirmed as being free of *D. gallinae* prior to the start of the experiment by a previously described method [18]. After 24 h collected from the laboratory culture, 220 (adult) mites were introduced to each cage by placing a Petri dish containing mites on the top of the cage [18]. There were three replicates of cages.

In order to address the animal welfare during the experiments, a total of 36 chicks were used. First, each cage was assigned with 6 chicks which were used in rotation; e.g. 3 of 6 chicks were replaced by 3 other ones every other day at the time of 1 h light, when most of mites were off-host. To prevent the possible development of immunity against *D. gallinae* and limit the negative effects of *D. gallinae* on the growth of the chicks [20], 6 chicks of each cage were then replaced by 6 new ones every 2 weeks. Behavioral reactions and physiological changes of chicks was monitored to evaluate the effects of the extended darkness and to ensure chicks were not exposed to too high levels of mites. The birds seemed not to be affected negatively by the extended darkness.

In order to clarify the possible effects on the population dynamics caused by replacing chicks during the experiment, we examined the mites on the body of chicks and the cages six times when performing chick replacement or while counting the mite. It was observed that most mites in the rearing system were hidden in traps, and only a small number of mites (less than 2.1%) were found on the body of chicks and the cages. The effects of lost mites on host and the cages on the population dynamics should therefore be negligible.

The number of mites in the traps was recorded weekly (at 7, 14, 21, 28 days) during the four-week trial. At each observation, all the traps were removed from the cages after 1 h light to ensure that most of *D. gallinae* were off-host. Total numbers of mites and eggs (including their feeding status and the number of dead individuals) dispersed on the mask were registered using a stereomicroscope (SteREO-Discovery.V12, ZEISS Microscopy) following a previously described method [18]. Mites were determined to have fed if they were engorged and/or had changed in color from pale grey or brown, to bright red. Mites were determined to be dead if neither light nor mechanical stimulation elicited an active movement of the legs. Survival and feeding rates of the recovered mites were calculated as follows: Survival rate (%) = Number of surviving mites/Total number of mite present in trap × 100%; Feeding rate (%) = Number of fed mites/Total number of mite present in trap × 100%. After counting, mites were moved back to the mask with a brush, and all the monitoring traps were affixed back at the predetermined positions in the cages.

The population dynamics of *D. gallinae* under lighting regimen of L:D of 1:23 h were compared with those under conventional lighting regimen of 12:12 h L:D, which had been carried out in a previous study [18].

### Engorgement, oviposition and hatchability

To study the effects of photoperiods on the fertility of *D. gallinae*, we assessed the oviposition of female mites and the hatchability of eggs under the two photoperiods of 12:12 h L:D and 1:23 h L:D, according to a previously described method [21]. At the end of the experiment of the population dynamics of *D. gallinae* under the 1:23 h L:D photoperiod, a random sample (*n* = 50) of engorged females from each cage was weighed using a Cubis® Analytical Balance (Sartorius Lab Instruments GmbH &Co. KG, Goettingen, Germany). As a comparison, another random sample (*n* = 50) of engorged females from the similar rearing system with photoperiod of 12:12 h L:D was collected and weighted immediately 0 days post-challenge (dpc). After weighing, individual mites were placed in wells of 96-well round bottomed ELISA plates right away to further evaluate their oviposition by counting the egg numbers produced by these mites. The plates were closed using plastic lids leaving a little gap that allowed air exchange but prevented mites from escaping. Plates were continuously kept in darkness at 30 °C, 75% RH for both 12:12 h L:D and 1:23 h L:D photoperiod. The numbers of eggs laid by each of the females were recorded, as well as the development of eggs. The mites were observed every 24 h for 7 days using a stereomicroscope and any dead mites were excluded from the experiment. The oviposition and hatchability were calculated on day 7. After observation, the mites were not placed back to the cage and were killed by freezing. The experiments were repeated in triplicate (3*n* = 3 × 50).

### Life-cycle of *D. gallinae*

In order to explore the effect of differing photoperiods on life-cycle of *D. gallinae*, duration of the complete *D. gallinae* life-cycle under two photoperiods of 12:12 h L:D and 1:23 h L:D, from the egg to a new egg generation, was both calculated and analyzed according to a previously described method with some modifications [22]. The conditions for the complete life-cycle for *D. gallinae* were unlimited food, 30 °C and 75% RH, with either of the two photoperiods of 12:12 h L:D or 1:23 h L:D. To obtain eggs for hatching success assessment, female *D. gallinae* were isolated in sealed petri dishes and allowed to oviposit during the oviposition period. The eggs were subsequently transferred into traps and installed to a new cage with new chicks. The initial cohort size was 300 eggs. The observations took place every 24 h for 10 days. At each observation, all traps were removed from the cage. The number of mites at different stages was counted under a stereomicroscope and the rate of mites at different stages was calculated every day. Observations continued until the mites reached the adult stage, and females started laying eggs. The time from the egg being deposited to a new egg generation was detected, which was recorded as the duration of the complete *D. gallinae* life-cycle.

### Statistical analysis

Since our experiments yielded three replicates per treatment (*n* = 3 cages) for each of the photoperiod, it was possible to perform statistical analyses. First, Kolmogorov–Smirnov and Leveneʼs test were conducted to test whether the data were normally distributed and homoscedastic. If data met the normal distribution and homogeneity of variance, Student’s t-test was performed to compare the number of mites and eggs, survival and feeding rate, weight, oviposition and hatchability, and life-cycle between conventional lighting regimen and prolonged darkness photoperiod. And if not, the nonparametric Mann–Whitney–Wilcoxon test was carried out. The significance level was set to *P* < 0.05. Data were expressed as mean ± standard deviation (SD).

## Results

### *cox*1 and ITS sequences

First, the size of the amplified PCR products of ITS and *cox*1 were confirmed by agarose gel electrophoresis, which was consistent with the expected size of ITS and *cox*1 gene fragment (Figure is not provided). Then, the ITS and *cox*1 region from three different individuals of *D. gallinae* were individually sequenced and edited in Contig Express (Vector Nti 11, Thermo Fisher, Waltham, USA). After sequence alignment, a single *cox*1 and ITS sequences from the different individuals were obtained. After performing BLAST search in GenBank (Megablast; https://blast.ncbi.nlm.nih.gov/Blast.cgi), there were several *D. gallinae* ITS fragments. They were identical to the 493 bp sequence generated from the strain used in our experiments with Genbank ID MK599419. These matches were identical to several GenBank entries in the alignment covering position 44–536 (relative to AM930886), and identical matches included several *D. gallinae* described from Japan (i.e. LC034921) [23] and France (AM930885) [24]. For *cox*1, our newly generated CPB-1 sequence (GenBank: MK599418) was identical with several *cox*1 sequences from mites from Asia in GenBank (including LC029544/Chiba_221 haplotype which had been reported as BJ1-haplotype recovered from Japan [23]); alignment was possible with this, from position 72–672 in relation our sequence (data not shown). These molecular data confirmed the identity of the mite used in the experiments to be *D. gallinae*.

### Temporal evolution of the population size

**Fig. 1:**
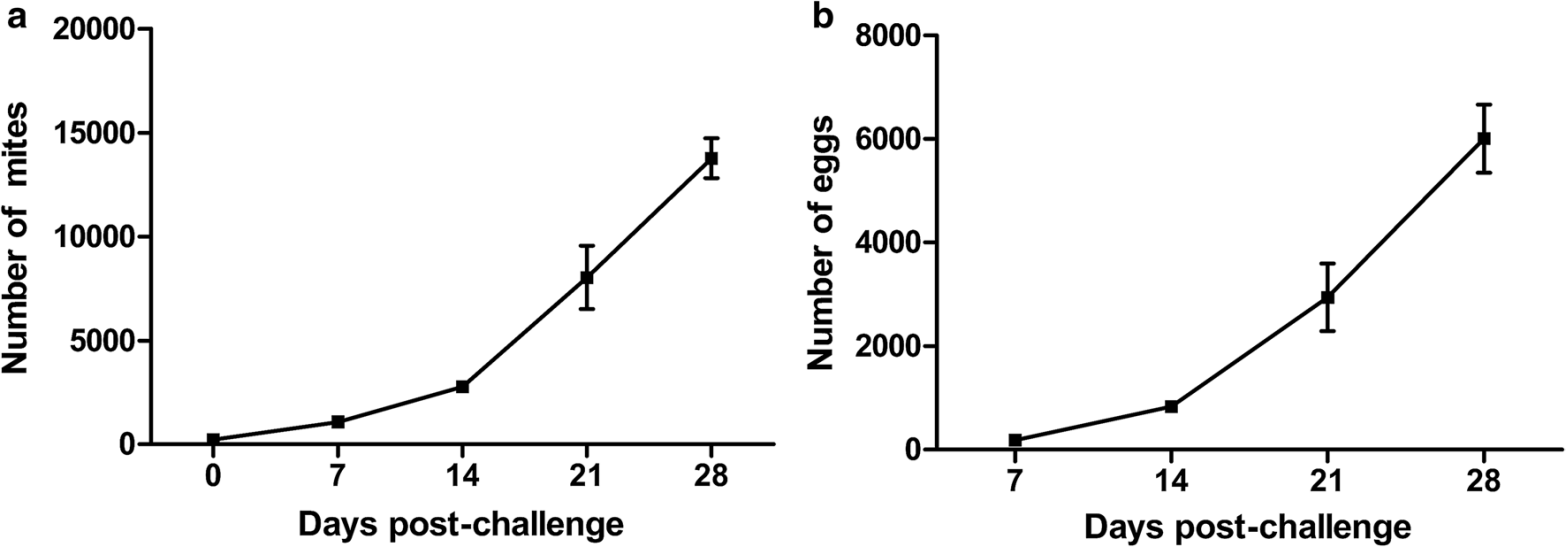
Total numbers of mites (**a**) and eggs (**b**) with a photoperiod of 1:23 h L:D. Chicks were challenged with 220 *D. gallinae* at 0 dpc and kept on prolonged darkness photoperiod of 1:23 h L:D. Total numbers of mites and eggs were counted under stereomicroscope at a magnification of 10× at 7, 14, 21 and 28 dpc. All values are shown as the mean ± SD from three independent experiments

Temporal evolution of the mite population size in the rearing system with photoperiod of 1:23 h L:D is presented in Fig. 1a. The data were compared with those from the groups subjected to 12:12 h L:D in our previous study [18]. All three replicates showed a remarkable increase of the *D. gallinae* population over a period of 4 weeks under prolonged darkness conditions since the introduction of the initial 220 mites to the rearing system. More in details, mite populations initially and slowly increased from 0 days to 14 dpc, thereafter the mite populations continuously and rapidly increased from 14 dpc. We observed that the number of mites under the photoperiod of 1:23 h L:D had a 62.6-fold increase over a period of 4 weeks with the mean number of 13,763 ± 956, whereas the number of mites under the photoperiod of 12:12 h L:D increased 24.7 times with the mean number from 220 to 5424 ± 317. The data indicate that the mite population under the photoperiod of 1:23 h L:D were extremely significantly higher (2.4-fold increase) than those under the photoperiod of 12:12 h L:D at 14 dpc (t-test, *t*_(4)_ = − 12.737, *P* < 0.001), 21 dpc (t-test, *t*_(4)_ = − 5.551, *P* = 0.005) and 28 dpc (t-test, *t*_(4)_ = − 14.331, *P* < 0.001), indicating that extending the period of darkness to 23 h effectively increased the population growth rate of *D. gallinae.*

Temporal evolution of the egg population size in the rearing system with photoperiod of 1:23 h L:D is shown in Fig. 1b. The number of eggs under two photoperiods was also compared. The data indicated that the increase of eggs under the photoperiod of 1:23 h L:D corresponded to mite population levels, with an increase of the mean number from 182 ± 31 to 6007 ± 657 (from 7 to 28 dpc), whereas the number of mites with a photoperiod of 12:12 h L:D indicated an increase from 59 ± 22 to 1652 ± 86. Additionally, the number of eggs under the photoperiod of 1:23 h L:D was extremely significantly higher (3.6-fold increase) compared with those under the photoperiod of 12:12 h L:D at 7 dpc (t-test, *t*_(4)_ = − 5.488, *P* = 0.005), 14 dpc (t-test, *t*_(4)_ = − 9.156, *P* = 0.001), 21 dpc (t-test, *t*_(4)_ = − 6.637, *P* = 0.003) and 28 dpc (t-test, *t*_(4)_ = − 11.392, *P* < 0.001).

### Survival and feeding rate

The survival rates of PRMs under the two photoperiods using our rearing system are presented in Table 1. We found that the mean survival rates for 1:23 h L:D fluctuated within a narrow range of 98.07 ± 0.10% to 98.93 ± 0.19% over the 4-week period, which were slightly higher than that under the 12:12 h L:D photoperiod, with the mean survival rates ranging from 95.88 ± 0.94% to 98.28 ± 0.33% at 7–28 dpc. These results overlap indicating that the survival rate of *D. gallinae* was not affected by the duration of darkness.

**Table 1:** Survival and feeding rates of *D. gallinae* with photoperiod of 1:23 h L:D

Variable	Photoperiod	Days post-challenge			
		7	14	21	28
Feeding rate (%)	1:23 h L:D	52.0 ± 7.0	36.7 ± 1.1	44.8 ± 5.9	50.5 ± 1.9
Survival rate (%)	1:23 h L:D	98.9 ± 0.6	98.9 ± 0.2	98.1 ± 0.1	98.6 ± 0.1

*Notes*: Chicks were challenged with 220 *D. gallinae* at 0 dpc and kept on prolonged darkness photoperiod of 1:23 h L:D. The numbers of dead and fed *D. gallinae* were recorded at 7, 14, 21 and 28 dpc. Survival and feeding rates were calculated as follows: Survival rate (%) = Number of surviving mites/Total number × 100%; Feeding rate (%) = Number of feeding mites/Total number × 100%. All values are shown as mean ± SD from three independent experiments

Overall feeding rates of protonymph, deutonymph and adult *D. gallinae* fed *in vivo* on chicks with both photoperiods are presented in Table 1. The mean feeding rates under photoperiod 1:23 h L:D, ranged from 36.7 ± 1.08% to 52 ± 7.02% over a period of 4 weeks. They were significantly higher than those observed under the photoperiod of 12:12 h L:D at 7 dpc (t-test, *t*_(4)_ = − 3.498, *P* = 0.025), 14 dpc (t-test, *t*_(4)_ = − 11.239, *P* < 0.001), 21 dpc (t-test, *t*_(4)_ = − 4.917, *P* = 0.008) and 28 dpc (t-test, *t*_(4)_ = − 9.223, *P* = 0.001) with the mean feeding rates ranging from 22.57 ± 1.89% to 37.30 ± 1.57%; indicating that extending the time of darkness to 23 h effectively improved the feeding rates of *D. gallinae.*

### Engorgement, oviposition and hatchability

To investigate whether prolonged darkness had the effect on the engorgement, oviposition and hatchability of female, fifty engorged female mites were collected and weighed. The weight of mites obtained in our study is presented in Table 2. The mean weight per female isolated from the rearing system with photoperiod of 1:23 h L:D in three batches was 0.26 ± 0.01 mg, which was extremely significantly higher than those (0.22 ± 0.01 mg) recovered from the rearing system with photoperiod of 12:12 h L:D (t-test, *t*_(4)_ = − 5.481, *P* = 0.005). The results indicate that the engorgement level of females under prolonged darkness was improved.

**Table 2:** The weight of females isolated from the rearing system under the two photoperiods

Photoperiod	No. of fed females	Total weight of mites (mg)	Mean ± SD (mg)	Mean weight/female (mg)
12:12 h L:D	50	10.37	10.90 ± 0.51	0.22 ± 0.01
	50	10.94		
	50	11.39		
1:23 h L:D	50	13.76	13.19 ± 0.51	0.26 ± 0.01
	50	13.02		
	50	12.78		

*Notes*: The traps in the rearing system under the two photoperiods were removed from the cages after 1 h light. Engorged females were selected randomly and weighted immediately (0 dpc)

The data on oviposition and hatchability of females isolated from the rearing system under two photoperiods are presented in Table 3. The mean number of eggs per female isolated from the rearing system with photoperiod of 12:12 h L:D were 3.46, 3.42 and 3.98 in three batches respectively, with an average of 3.62 ± 0.31. In contrast, the mean number of eggs per female separated from the rearing system with photoperiod of 1:23 h L:D were 6.04, 6.12 and 5.46 in three batches respectively, with an average of 5.87 ± 0.36, which was extremely significantly higher than that in the rearing system with photoperiod of 12:12 h L:D (t-test, *t*_(4)_ = − 8.186, *P* = 0.001).

**Table 3:** Oviposition and hatchability of *D. gallinae* in the rearing system under the two photoperiods

Photoperiod	Females	Oviposition			Hatchability		
		Total no. of eggs	Mean no. of eggs/female	Mean ± SD	Number	Percentage	Mean ± SD
12:12 h L:D	50	173	3.46	3.62 ± 0.31	168	97.11	97.45 ± 0.70%
	50	171	3.42		168	98.25	
	50	199	3.98		193	96.98	
1:23 h L:D	50	302	6.04	5.87 ± 0.36	299	99.0	97.93 ± 0.92%
	50	306	6.12		298	97.4	
	50	273	5.46		266	97.4	

*Notes*: Engorged females in the rearing system under two photoperiods were selected randomly and placed individually in each well of 96-well round bottomed ELISA plates. The numbers of eggs laid by each female were recorded as well as the development of eggs. The experiments were repeated in triplicate. The mites were observed every 24 h for one week. Oviposition and hatchability were calculated at day 7

Hatchability of eggs laid by female isolated from the rearing system with two photoperiods was both above 96% as shown in Table 3, indicating high egg viability. Furthermore, the mean hatchability with photoperiod of 12:12 h L:D was 97.45 ± 0.70%, which was slightly lower than those in the rearing system with photoperiod of 1:23 h L:D, indicating an egg hatchability of 97.93 ± 0.92% (t-test, *t*_(4)_ = − 0.728, *P* = 0.507). The similar egg viability values observed for both photoperiods indicate that prolonged darkness does not interfere with the success of mite egg development.

### Life-cycle time of the mites

To further study the effect of darkness on the life-cycle time of *D. gallinae*, a 10-days study on the life-cycle time under the two photoperiods of 12:12 h L:D and 1:23 h L:D was conducted using the rearing system. As shown in Table 4, the minimum generation time of *D. gallinae* under the two photoperiod conditions, from the egg to a new egg, both were 9 days, indicating that duration of the life-cycle was not influenced by prolonged darkness. We found that eggs kept at the two photoperiods had a similar developmental period (2 days). Duration of the larval under two photoperiods was 1 day. Besides, protonymph emerged at 2 dpc, and began to disappear at 6 dpc, demonstrating most of protonymph completed development in 5 days. The quickest appearance of deutonymphal stages in both photoperiod conditions was on day 5, followed by adult emerging at 8 dpc. The adult started feeding on chicks and followed by egg laying at 9 dpc. These results indicate that the duration of the complete *D. gallinae* life-cycle under the two photoperiod conditions were similar. It is therefore demonstrated in these experiments that prolonged darkness had little, or no measurable effect on the rate of the life-cycle of *D. gallinae*.

**Table 4:** The proportion of each developmental stage of life-cycle for *D. gallinae* in the rearing system under the two photoperiods (Mean ± SD)

Dpc	12:12 h L:D					1:23 h L:D				
	Eggs	Larvae	Protonymphs	Deutonymphs	Adults	Eggs	Larvae	Protonymphs	Deutonymphs	Adults
1	100	0	0	0	0	100	0	0	0	0
2	0	23.20 ± 8.60	76.80 ± 8.60	0	0	0	17.87 ± 3.40	82.13 ± 3.40	0	0
3	0	0	100	0	0	0	0	100	0	0
4	0	0	100	0	0	0	0	100	0	0
5	0	0	100	0	0	0	0	100	0	0
6	0	0	10.87 ± 6.59	89.13 ± 6.59	0	0	0	75.67 ± 18.54	24.33 ± 18.54	0
7	0	0	6.90 ± 4.37	93.10 ± 4.37	0	0	0	52.80 ± 26.17	47.20 ± 26.17	0
8	0	0	2.20 ± 2.25	93.73 ± 1.10	4.07 ± 1.22	0	0	23.73 ± 10.23	73.87 ± 8.36	2.40 ± 2.61
9	0.83 ± 0.06	0	0	78.60 ± 3.82	20.57 ± 3.87	2.70 ± 4.68	0	3.20 ± 3.87	77.47 ± 6.87	16.63 ± 3.40
10	3.33 ± 0.96	0	0	51.37 ± 9.50	45.30 ± 8.55	7.83 ± 3.51	0	0	58.53 ± 10.02	33.63 ± 10.75

*Notes*: The complete life-cycle of *D. gallinae*, from the egg to a new egg generation, was carried out using the in *vivo* feeding system. The initial cohort size was 300 eggs. The observations took place every 24 h for 10 days. At each observation, the number of mites at different stages was counted under a stereomicroscope and the rate of mites at different stages was calculated every day. Observations were continued until the mites had reached the adult stage and laid the first egg

## Discussion

*Dermanyssus gallinae* parasites tend to attack hens at night and remain hidden during the day. Due to this behaviour, many farmers and researchers have become interested in using photoperiods to keep the mite populations in hiding, and thus limit their attacks on hens [16]. Studies have shown that in the presence of birds, the light in the presence of CO_2_ might make mite freeze up and stand still for a period of time, which may be a behavior to avoid predation by birds [14]. This is the possible reason for *D. gallinae* generally taking blood meal at night. To develop into next stage, several phases of the *D. gallinae*’s life-cycle require blood meals. For example, larval and nymphal mites have to feed in order to moult and the adult female mite needs a blood meal before lying each batch of eggs [25]. Thus, a high feeding rate is conducive to rapid reproduction of mites and reduction of feeding rate of *D. gallinae* can be an optional method for control of *D. gallinae*. Several authors previously worked on *in vitro* blood meals for *D. gallinae*, finding that temperature, light and relative humidity influence feeding rates, as well as the duration of starvation and the temperature where mites were kept at during starvation (i.e. [26, 27]). However, the detailed effects of darkness/light on the population dynamics, the survival and feeding rates, oviposition, and the egg hatchability, as well as the duration of life-cycle of *D. gallinae* were not well understood.

The study suggested that under prolonged dark conditions, there was a marked increase of mite population levels, compared with the normal light regime, e.g. on 28 dpc the mean mite number of 13,763 *versus* 5424 and the mean egg number of 6007 *versus* 1652 (Fig. 1 and [18]).

To clarify the possible mechanism leading to increase of mite populations under prolonged darkness, several key factors were evaluated, including survival and feeding rate, the duration of stages and the life-cycle, engorgement, oviposition and hatchability. The results in the experiments indicate that two factors, increased feeding rate of mite and ovipositioning in the mite were both associated with an increased population of *D. gallinae* in prolonged darkness (1:23 h L:D). More specifically, the overall feeding rates of protonymph, deutonymph and adult *D. gallinae* in this group ranged from 52.0 ± 7.0% to 36.7 ± 1.1%, significantly higher than that of mite under conventional light conditions (22.6 ± 1.9% to 37.3 ± 1.6%) (12:12 h L:D) [18]. It seemed that prolonged darkness facilitated feeding of the mites. These observations confirm that *D. gallinae* had a feeding behavior which was subject to a photoperiodic influence [5]. These results are inconsonant with those from Kirkwood [13], who investigated a possible preconditioning effect of light on feeding. In his study, mites were starved for 1, 2, 3 and 6 weeks in natural alternations of daylight and darkness, in continuous light or in continuous darkness, and then they were allowed to feed at night in the dark. He found that no difference was recorded between the number of mites which fed after exposure to continuous light (74.7%) and those which fed after exposure to continuous darkness (74.0%) [13]. He claimed that *D. gallinae* does not have a feeding rhythm, which is subject to a photoperiodic influence. However, it is notable that in the present study the feeding rate of mites was observed in conditions where mites could repeatedly take blood meals without preconditioning of starvation. Our results suggest that darkness were more likely to stimulate mites reproduction, than the presence of light, which is in accordance with results obtained on layer farms by Sokół et al. [16].

Another reason for the fact that prolonged darkness significantly promoted the population growth of *D. gallinae* was increased oviposition of *D. gallinae* observed in this group. Oviposition of *D. gallinae* is sustained until all blood ingested (red color) has been digested, ending when the mite become gray [28]. In the present study, the mean weight of females with photoperiod of 1:23 h L:D was higher than that of 12:12 h L:D (0.26 *vs* 0.22 mg), indicating an increased level of engorgement under prolonged dark conditions. Interestingly, the total mean number of eggs from three separate batches with photoperiod of 1:23 h L:D (5.87) was also significantly higher than that of the other group (3.62). These observations suggest that the mean number of eggs produced by *D. gallinae* is positively related to the weight of engorged females. Blood meals provide the protein required for egg maturation in some medically important insects (e.g. mosquitoes) [29]. In mosquitoes, it has been found that the blood-meal intake and physiological aging have considerable effects on egg production [30, 31]. It has also been demonstrated that the number of eggs produced by ticks increases roughly linearly with increasing weight of an engorged female [32]. However, it should be mentioned that the huge differences existed in the oviposition cycles between ticks (perform a single oviposition cycle) and *D. gallinae* (up to 8 oviposition cycles). Like *D. gallinae*, most mosquitoes perform several oviposition cycles. And the oviposition status (strongly related to age) of *D. gallinae* was also strongly related to the oviposition level [33]. Many adult females of *D. gallinae* did not oviposit after their first blood meal and required an additional feeding before laying eggs. The mean number of eggs produced per ovipositing female gradually increased after first blood meal, reaching the peak after the third, fourth and fifth feedings and decreased subsequent to the fifth feeding. Interestingly, in the present study, the difference in the weight of female and the mean number of eggs laid by female was so much consistent with different age means (lighter females laid less than 4 eggs, whilst heavier ones laid more than 6 eggs), which may result from different oviposition statuses (nulliparous, uniparous, … multiparous) in the sampled females. This also likely resulted in a significant increase of the population growth rate. Unfortunately, since the oviposition statuses of *D. gallinae* were not determined in the present study, their definite effects on the population growth of *D. gallinae* need to be further studied.

The duration of the complete *D. gallinae* life-cycle under the two different light regimes conditions, was 9 days. These results are in agreement with those reported by other authors in trials conducted at temperatures between 25–28.3 °C. Wood [34] observed that the life-cycle of *D. gallinae* from engorged female to a new adult generation ranged from 8.5 to 10 days, Sikes & Chamberlain reported 8–9 days [9], Harrison 7–10 days [35], and Tucci 7.9 days [36], indicating that the results obtained herein demonstrate that prolonged darkness had little or no observable effect on the longevity of *D. gallinae* life-cycle.

It should be noted that the difference of the ability of the parasite to multiply may be influenced not only by physical factors such as light, temperature, humidity, but also biotic factors such as availability of hosts, or predation. Increased light may also increase the predation on mite by chickens, which have been shown to peck on mite [12]. It is possible that the predation levels on mite by chickens, were higher in the groups where more light was present (12:12 h L:D) and could limit mite growth compared to the group in prolonged darkness.

Physical control of *D. gallinae* incorporates measures such as manual layer house cleaning, temperature manipulation, and treatment using dust or other chemical compounds as well as light manipulation. Light manipulation could be considered as a promising tool for the control of *D. gallinae*, which has less problems concerning resistance and residues as seen in the using of chemicals. The basic knowledge of the effects of lighting patterns on *D. gallinae* is a first step towards the development of control strategy of *D. gallinae* by light manipulation. In the present experiment, we focused on changing the light regimes and evaluated the effect on the mite population. The present study demonstrated that prolonged darkness significantly promoted the population growth of *D. gallinae*, indicating an application potential in the control of *D. gallinae* by light manipulation. However, how to employ light manipulation in practice on layer farms to control *D. gallinae* is another story. Welfare of animals and regulations should be taken into account when the control strategy is applied in practice. For example, current EU legislation indicates that lighting regime must follow a 24-hour rhythm and include an adequate uninterrupted period of darkness lasting, about one third of the day (8 h), so that the hens may rest and to avoid problems such as immunodepression and ocular anomalies. One possible solution to this problem is to apply intermittent lighting during normal light periods, which is with (presumably) fewer welfare implications [5].

## Conclusions

Our findings demonstrated that prolonged darkness could significantly increase the feeding rate, engorgement level and oviposition of *D. gallinae*, resulting in the significantly increased number of mites and eggs in the rearing system, e.g. promoted population growth of *D. gallinae*. The present study provided an inspiration point for rapid reproduction of mites under the laboratory conditions that the optimized light regimen could remarkably promote the population growth of *D. gallinae*. The present study also shed a light for development of control strategy of *D. gallinae via* changing lighting rhythms. However, animal welfare and regulations should be seriously taken into account when this strategy is used on layer farms.

## Abbreviations

dpc: days post-challenge
RH: relative humidity
12:12 L:D: 12 h light:12 h darkness photoperiod
1:23 L:D: 1 h light:23 h darkness photoperiod
